# A compilation of life cycle studies for six household detergent product categories in Europe: the basis for product-specific A.I.S.E. Charter Advanced Sustainability Profiles

**DOI:** 10.1186/s12302-015-0055-4

**Published:** 2015-10-05

**Authors:** Laura Golsteijn, Rimousky Menkveld, Henry King, Christine Schneider, Diederik Schowanek, Sascha Nissen

**Affiliations:** 1 PRé Consultants bv, Stationsplein 121, 3818 LE Amersfoort, The Netherlands; 2 Unilever, Safety and Environment Assurance Centre, Sharnbrook, Bedford, MK44 1LQ UK; 3 Henkel AG & Co. KGaA, Henkelstrasse 67, 40589 Düsseldorf, Germany; 4 Procter & Gamble, Brussels Innovation Centre, Temselaan 100, 1853 Strombeek-Bever, Belgium; 5 International Association for Soaps, Detergents and Maintenance Products (A.I.S.E.), Boulevard du Souverain 165, 1160 Brussels, Belgium

**Keywords:** Cleaning agent, Life cycle assessment (LCA), Environmental impact, Sustainable use, Consumer information, Resource efficiency, Industry progress

## Abstract

**Background:**

A.I.S.E., the International Association for Soaps, Detergents and Maintenance Products, launched the ‘A.I.S.E. Charter for Sustainable Cleaning’ in Europe in 2005 to promote sustainability in the cleaning and maintenance products industry. This Charter is a proactive programme for translating the concept of sustainable innovation into reality and actions. Per product category, life cycle assessments (LCA) are used to set sustainability criteria that are ambitious, but also achievable by all market players.

**Results:**

This paper presents and discusses LCAs of six household detergent product categories conducted for the Charter, i.e.: manual dishwashing detergents, powder and tablet laundry detergents, window glass trigger spray cleaners, bathroom trigger spray cleaners, acid toilet cleaners, and bleach toilet cleaners. Relevant impact categories are identified, as well as the life cycle stages with the largest contribution to the environmental impact.

**Conclusions:**

It was concluded that the variables that mainly drive the results (i.e. the environmental hotspots) for manual dishwashing detergents and laundry detergents were the water temperature, water consumption (for manual dishwashing detergents), product dosage (for laundry detergents), and the choice and amount of surfactant. By contrast, for bathroom trigger sprays, acid and bleach toilet cleaners, the driving factors were plastic packaging, transportation to retailer, and specific ingredients. Additionally, the type of surfactant was important for bleach toilet cleaners. For window glass trigger sprays, the driving factors were the plastic packaging and the type of surfactant, and the other ingredients were of less importance. A.I.S.E. used the results of the studies to establish sustainability criteria, the so-called ‘Charter Advanced Sustainability Profiles’, which led to improvements in the marketplace.

**Electronic supplementary material:**

The online version of this article (doi:10.1186/s12302-015-0055-4) contains supplementary material, which is available to authorized users.

## Background

A wide variety of cleaning products is used in European households and industrial and institutional sectors. A.I.S.E., the International Association for Soaps, Detergents and Maintenance Products, has developed and implemented a ‘Charter for Sustainable Cleaning’ (hereafter the ‘Charter’) in Europe [1]. Launched in 2005, this voluntary initiative is a comprehensive life cycle-based framework for promoting a common industry approach regarding sustainability. The Charter covers a wide variety of activities and requirements, ranging from the human and environmental safety of chemicals and products, to occupational health and safety, resource use, and consumer information. The two main goals are to promote continuous efforts of the whole industry to design and make their products more sustainable and to encourage consumers to adopt more sustainable ways of doing their washing, cleaning, and household maintenance.

**Fig. 1:**
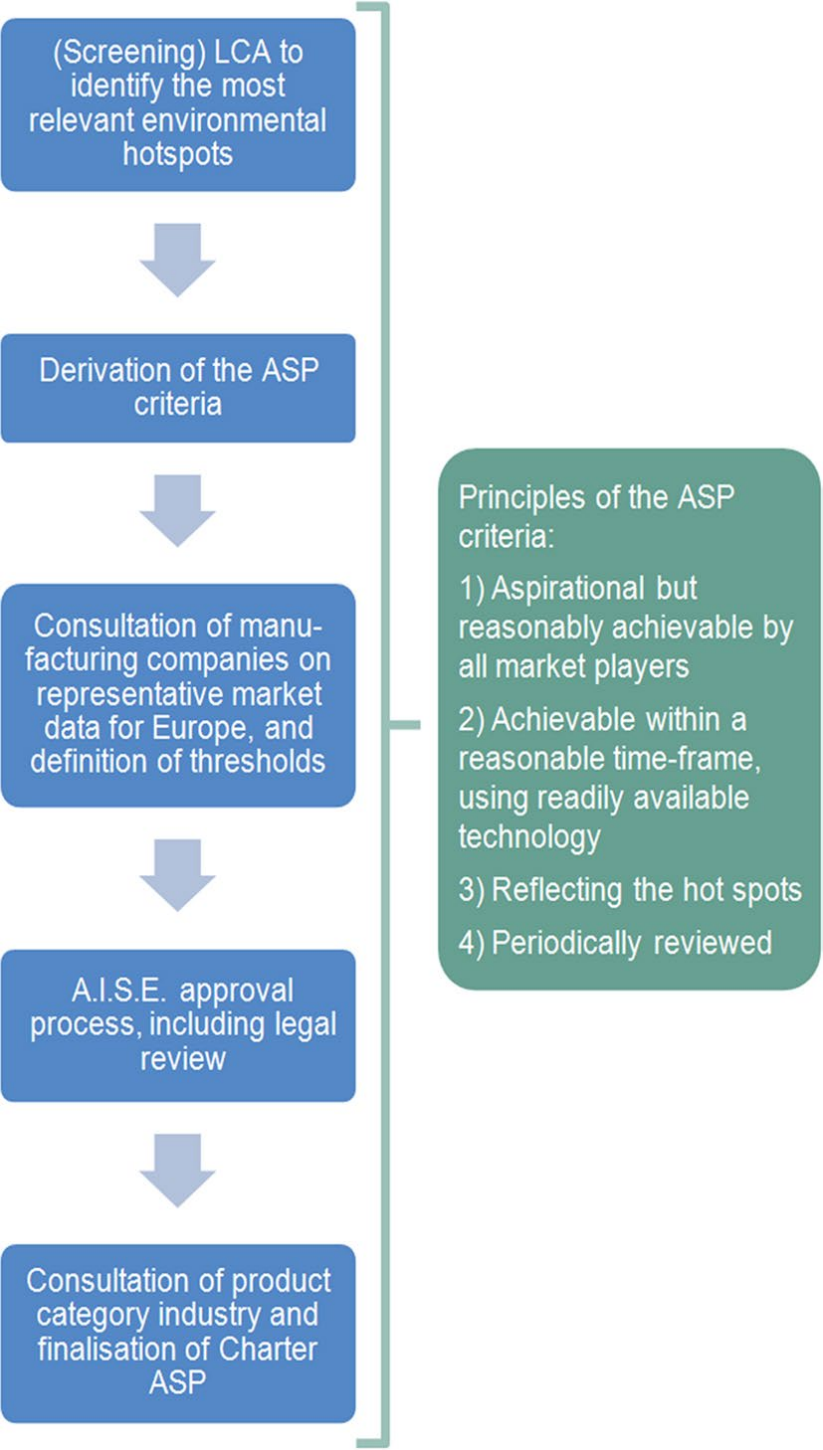
Principles and stepwise process to derive Charter ASP criteria, based on LCA

**Fig. 2:**
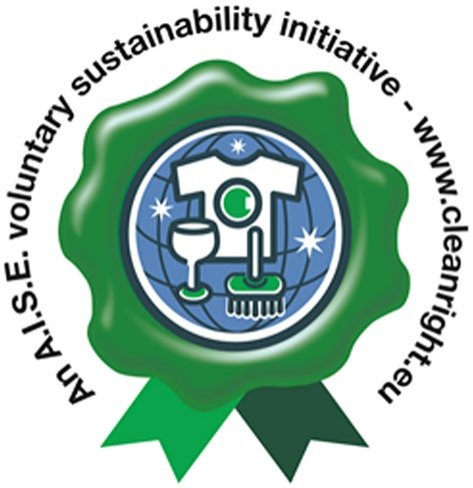
Product logo of the Charter for sustainable cleaning, indicating that the product meets the Charter ASP criteria

In October 2010, A.I.S.E. published the “Charter Update 2010”. A key component of this update is the addition of a ‘product dimension’, which further strengthens the Charter by enabling it to more completely cover the whole life of a product in terms of sustainability, from material sourcing to product manufacturing and end use. For major product categories, Advanced Sustainability Profiles (ASPs) are created [2]. The ASPs are used to define a set of criteria and thresholds that a product must meet to improve the environmental performance. Figure 1 provides an overview on the principles and stepwise process to derive Charter ASP criteria. The accompanying logo signals to consumers that a product fulfils those criteria and thresholds (see Fig. 2).

As a quantitative basis for selecting the ASP criteria, life cycle assessments (LCAs) were performed between 2011 and 2013 to identify the most relevant environmental impacts per product category. This paper presents the results of the LCAs for six product categories: manual dishwashing detergents, powder and tablet laundry detergents, window glass trigger spray cleaners, bathroom trigger spray cleaners, acid toilet cleaners, and bleach toilet cleaners. Automatic dishwashing detergents where not subject to these LCAs, because company data were available. These were used to derive the Charter ASPs for this product category. It (a) identifies the life cycle stages with the largest contribution to the environmental impact; (b) assesses which impact categories are relevant for the different product groups by applying a normalisation approach; (c) identifies the variables that mainly drive the results per product group; and (d) discusses the practical implications of the LCA results for the assessment of products on the market and for the design of products via the development of relevant ASP criteria.

## Methods

### Goal and scope

#### Functional unit and reference flow

The overall objective of the studies was to identify the most relevant impact categories and environmental hotspots of the life cycle per product category, rather than absolute results. Product usage data were provided by A.I.S.E. member companies. The functional units were defined as a ‘typical application’ of the product based on recommended dosages or expert opinion. Table 1 shows the functional unit and reference flow for each household detergent product category.

**Table 1:** Functional unit and reference flow for the studied household product categories

Product category	Functional unit	Reference flow^a^
Manual (hand) dishwashing detergent	The manual washing of four place settings^b^	8 ml of detergent for the full sink scenario, and 12 ml for the direct application scenario^c^
Compact powder and tablet laundry detergent	1 normally soiled wash^d^	81.5 g of compact powder, or a powder tablet of 63.8 g
Window glass trigger spray	1 m^2^ of a window or glass surface manually wetted and cleaned	10 ml of the product^e^
Bathroom trigger spray	1 m^2^ of a bathroom surface manually wetted and cleaned	10 ml of the product^f^
Acid toilet cleaner	Cleaning one toilet bowl	50 ml of liquid acid toilet cleaner
Bleach toilet cleaner	cleaning one toilet bowl	80 l of liquid bleach cleaner

^a^All reference dosages were provided by A.I.S.E., based on experts’ opinion, taking into account the current market situation and consumer habits

^b^Four complete sets of dishes and cutlery provided for one person at a meal [5, 6]

^c^For manual dishwashing, two scenarios were assessed, i.e. the full sink scenario in which the sink is filled up with water first and the dishes are washed but not rinsed, and the direct application scenario in which the tap runs while washing and rinsing, for at least part of the time. In both scenarios, drying of the place settings was excluded

^d^Average load (5 kg) of normally soiled cloth using medium hardness water in a 6 kg machine and with a reference wash temperature of 40 °C [14, 19]

^e^We assumed that 6.25 squirts are required to wet a surface of 1 m^2^. On average, one trigger dose releases 1.6 ml

^f^To wet a surface of 1 m^2^, five trigger doses are required. One trigger dose releases on average 1.2–2 ml of the product

#### System boundaries

**Fig. 3:**
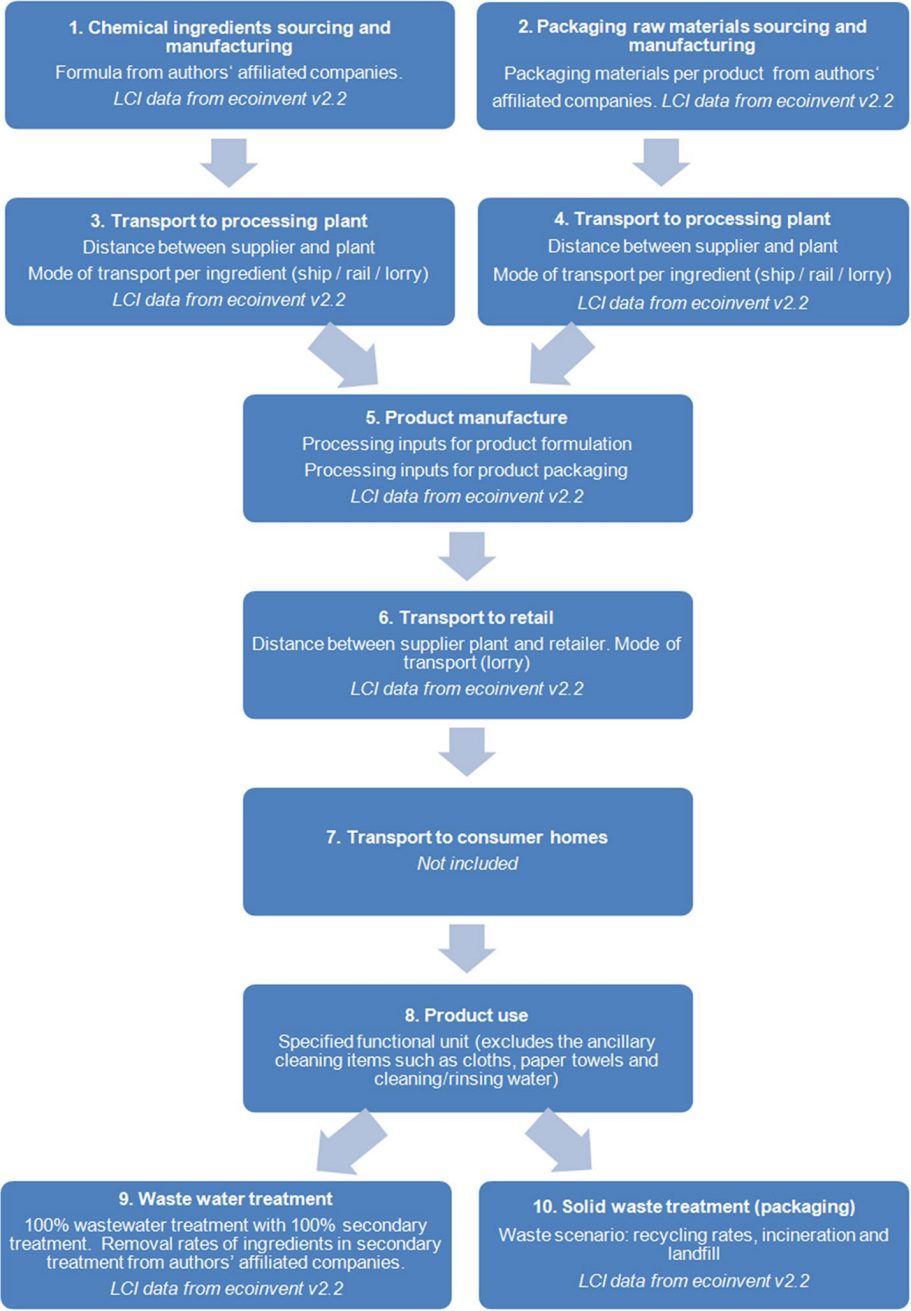
System model and data inventory (with regard to ‘9. Waste water treatment’, human and environmental toxicity are being addressed via risk assessment methods)

The product categories were analysed in cradle-to-grave LCAs for representative products sold in the EU during the period of 2011 and 2013. A schematic overview of the system boundaries is given in Fig. 3. We included sourcing of the ingredients and packaging materials (processes 1–4), manufacture and packing of the product (5), distribution to retail (6), consumer use of the product (8), product and packaging disposal, and wastewater treatment (9–10).

Storage in retail was omitted, as well as transport from retail to consumer homes (7), because product and market-specific data were unavailable. However, studies for other categories show that these impacts are generally minimal when compared with other activities and typical shopping habits. Capital goods on background and foreground data and provision of tap water to the home were also excluded from the scope of the study where possible.

The environmental impacts associated with the two scenarios of manual dish washing of four place settings included the amount of water used and the energy to heat the water. The environmental impacts associated with washing of clothes include the amount of water used in an average front-loading machine load and the energy to heat the water. The dosing device used for the compact powder laundry detergent is not included. In the use phase of both trigger sprays, the use of a cloth, paper, or other implement to wipe the surfaces was excluded, as the focus of the LCA is on the product itself. For both types of toilet cleaners, the environmental impacts associated with the flush water were excluded, since this is not part of the product use instructions.

For packaging disposal, we applied the recycled content method. This means that the benefits and burdens associated with recycling fell outside the scope of the study. The remaining waste, sent to landfill and incineration instead of recycled, was allocated to the product itself.

Environmental and human safety aspects are out of scope and are addressed through risk assessment in the Charter scheme. To qualify for the Charter ASP logo, products must successfully pass an environmental safety check (ESC [3]), which means that all ingredients have to be below a no-effect limit for aquatic toxicity. The ESC tool performs calculations based on the core concept of risk assessment: it conservatively projects predicted environmental concentrations (=PEC) for the environment and compares these to relevant predicted no-effect concentrations (=PNEC). The result is expressed as a projected environmental safety ratio (PEC/PNEC = PESR). To pass the ESC check, the PESR for each ingredient as formulated and dosed must be less than 1. This corresponds to the PEC/PNEC <1 criterion which is the basis for concluding no significant risk of adverse effects in the REACh legislation.

#### Data collection and assumptions

The Ecoinvent v2.2 database was used for all background processes [4]. This includes data on emissions, raw material inputs, technology, energy inputs, water, and transport. Figure 3 shows an overview of the system modelled and the additional datasets provided.

Data on the different product categories including product and packaging specifications consumer use, and end of life were provided by A.I.S.E member companies. Per product category, a table with the key assumptions is available in Additional file 1.

For all product categories, typical transport distances and modes (transport to manufacturing and transport to retailer) were provided by A.I.S.E. The transport of ingredients to the product manufacturing site was assumed to be 8000 km by boat for the renewable part of the surfactants and 2000 km by train for the other ingredients. The transport to retail was assumed to be 1200 km by lorry.

Excluding water from the product composition, some ingredients from the inventory list did not have specific LCI data available and required a proxy. This was roughly 24 % of the mass balance of the product formulation for manual dishwashing detergents, 9 % for window glass trigger spray cleaners, 10 % for bathroom trigger spray cleaners, 15 % for acid toilet cleaners, and 20 % for bleach toilet cleaners (see also Additional file 1 for more details). For compact powder and tablet laundry detergent, this was not the case. As no proxies were available for dye and fragrance, these were modelled as empty processes. Data on the composition of a typical manual dishwashing detergent (e.g. formulation and packaging specifications), product manufacturing, and use were provided by Procter & Gamble. For compact powder and tablet laundry detergents, these data were provided by Henkel, Procter & Gamble, and Unilever. For window glass trigger spray cleaners and acid toilet cleaners, these data were provided by Henkel. And for bathroom trigger spray cleaners and bleach toilet cleaners, these data were provided by Unilever.

For manual dishwashing, the specifications of a place setting were taken from Stamminger et al. [5, 6]. The water temperature for dish washing was based on the maximum temperature people can stand comfortably with bare hands (45 °C). For laundering, the wash temperature was set to 40 °C [7].

The recycling rates for paper, board, and plastic were taken from Eurostat [8].

### Impact assessment

A comprehensive set of environmental impact categories was investigated to identify the most relevant impact categories for the product. The selected method was the internationally recognised Life Cycle Impact Assessment (LCIA) method ReCiPe [9]. This method assesses 18 different impacts categories (midpoint level), which can subsequently be aggregated into three damage categories (end point level). The present study reports 12 impact categories at the midpoint level, excluding environmental and human toxicity impacts from the assessment (see “System boundaries”). Additional file 1: Figure S1 in the appendices shows the relationship between the inventory data and impact indicators.

Normalisation at end point was used to identify the relative size of the impact categories and the relevance for the overall damage to human health, ecosystem quality, or resource depletion. The results were calculated based on ReCiPe end point [9], using the so-called hierarchist perspective with European normalisation data from the year 2000 [10]. The weighting set used is human health (40 %), ecosystem quality (40 %) and resource depletion (20 %), which reflects the views of the European society [11].

In addition to the impact categories identified in ReCiPe, the cumulative energy demand (CED) per product category was assessed. The method to calculate the CED (v1.08) is based on the method published by ecoinvent version 2.0 and expanded by PRé Consultants for energy resources available in the SimaPro database [12]. Characterisation factors are given for the energy resources divided into five impact categories: nonrenewable, fossil; nonrenewable, nuclear; renewable, biomass; renewable, wind, solar, geothermal; and renewable, water. Normalisation is not part of this method.

## Results

**Fig. 4:**
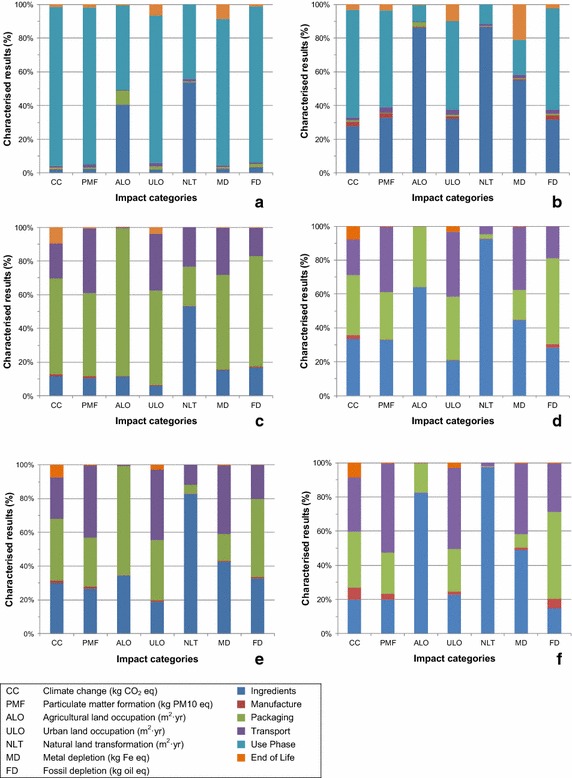
Characterised midpoint results per product category: **a** manual dishwashing detergent (full sink approach), **b** tablet laundry detergent, **c** window glass trigger spray, **d** bathroom trigger spray, **e** acid toilet cleaner, and **f** bleach toilet cleaner

**Fig. 5:**
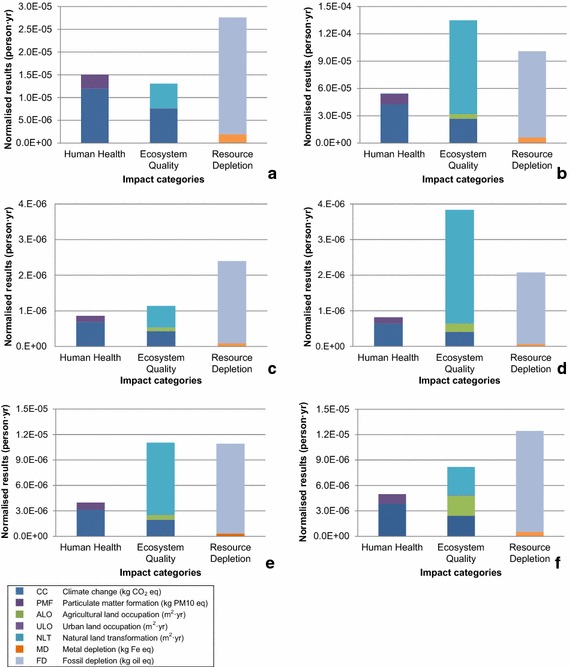
Normalised end point results per product category: **a** manual dishwashing detergent (full sink approach), **b** tablet laundry detergent, **c** window glass trigger spray, **d** bathroom trigger spray, **e** acid toilet cleaner, and **f** bleach toilet cleaner

Figure 4 shows the contribution of the different life cycle stages to the environmental impacts per product category, using characterised midpoint results from ReCiPe. The results shown here are only for the impact categories that were found to be important by normalisation (see Fig. 5). For the results of all impact categories, please see Figure S2 of the Additional file 1. Table 2 shows the life cycle stage contribution to the total cumulative energy demand of the product categories. Figure 5 shows the normalised values of the endpoint indicators per product category.

**Table 2:** Contributions of the life cycle stages to the total cumulative energy demand per product category (one typical application)

Product category	Total (MJ)	Ingredients	Manufacture	Packaging	Transport	Use phase	End of life
Manual dishwashing detergent (full sink)	3.70	0.01	0.10	0.05	0.02	3.49	0.03
Manual dishwashing detergent (direct application)	4.25	0.01	0.15	0.05	0.04	3.94	0.06
Tablet laundry detergent	13.0	3.70	0.38	0.10	0.19	8.35	0.24
Compact powder laundry detergent	12.2	2.84	0.38	0.15	0.27	8.35	0.24
Window glass trigger spray	0.24	0.04	<0.005	0.16	0.03	n.a.	<0.005
Bathroom trigger spray	0.22	0.08	<0.005	0.10	0.03	n.a.	<0.005
Acid toilet cleaner	1.09	0.36	0.01	0.52	0.19	n.a.	<0.005
Bleach toilet cleaner	1.28	0.28	0.08	0.63	0.30	n.a.	<0.005

### Manual dishwashing detergent

#### Environmental impact of the life cycle stages

The results for manual dishwashing with a full sink approach are shown in Fig. 4 and Table S27 **(**Additional file 1). The results for a direct application approach showed similar trends (see Table S28 of the Additional file 1). The life cycle stage with the largest contribution to the environmental impact of a manual dishwashing detergent was the use phase, in particular the energy needed to heat the water during manual dishwashing. The use phase contributed 86–98 % for all impact categories with the exception of natural land transformation (44 %) and agricultural land occupation (50 %) which were driven by the ingredients sourcing, in particular the surfactant. The surfactant modelled in this study was of an oleochemical origin (palm or coconut), which had an effect on natural land transformation.

Product manufacture, packaging, transport, and the end of life had a minor contribution towards all the impact categories in comparison to the use phase and ingredients sourcing.

The cumulative energy demand was 3.70 MJ for the full sink approach and 4.25 MJ for direct application (see Table 2). In both cases, the major part (3.49 and 3.94 MJ, respectively) could be attributed to the energy needed to heat the water during manual dishwashing (7.5 l of water for the full sink approach and 15 l for direct application).

#### Identification of relevant impact categories

For a manual dishwashing detergent, the most relevant impact categories relative to the reference were fossil depletion, climate change (the effect on both human health and ecosystem quality), particulate matter formation, and natural land transformation (see Fig. 5). The impacts on climate change, fossil depletion, and particulate matter formation were interrelated, and driven by the same factors, in this case the energy use during the use phase. Impacts on natural land transformation were mainly driven by the surfactant.

### Compact powder and tablet laundry detergents

#### Environmental impact of the life cycle stages

The results for a tablet laundry detergent are shown in Fig. 4 and Table S29 of Additional file 1. The results for a compact powder laundry detergent showed a similar trend (see Table S30 of the Additional file 1). The life cycle stage with the largest contribution to the overall environmental impact was the use phase, in particular, the energy needed to heat the water during the wash cycle. The impact caused ranged from 46 to 95 % in most impact categories, with the exception of four impact categories which were driven by the ingredients sourcing.

The impacts on agricultural land occupation (86 %) and natural land transformation (86 %) were due to the surfactant choice. The surfactant modelled in this study was of a mixed origin [i.e. both oleo chemical origin (palm and coconut resources) and petrochemical], and the fraction of oleo chemicals drives the impact categories for natural land transformation and agricultural land occupation. Marine eutrophication (59 %) and metal depletion (55 %) impacts were primarily due to the builders used in the frame formula.

Compared to the use phase, manufacture, packaging, transport, and the end of life had a minor contribution towards the total environmental impact of laundry detergents.

Table 2 shows that the cumulative energy demand of a tablet laundry detergent is 13.0 MJ, of which the major part (8.35 MJ, i.e. 64 %) could be attributed to the use phase. For a compact powder laundry detergent, the cumulative energy demand was 12.2 MJ, with also 8.35 MJ (68 %) attributable to the use phase.

#### Identification of relevant impact categories

For solid laundry detergents (powder and tablet), the most important impact categories relative to the reference (i.e. average impacts of a European citizen in the year 2000) were fossil depletion, climate change (the effect on both human health and ecosystem quality), particulate matter formation, and natural land transformation (see Fig. 5). The impacts on climate change, fossil depletion, and particulate matter formation were interrelated and driven by the same factors, namely fossil energy use. Impacts on natural land transformation were mainly driven by the feedstock (source) of the surfactant.

### Window glass trigger spray

#### Environmental impact of the life cycle stages

For a window glass trigger spray, packaging had the largest contribution on agricultural land occupation (88 %), ionising radiation (77 %), freshwater eutrophication (75 %), fossil depletion (65 %), climate change (57 %), urban land occupation (56 %), and metal depletion (56 %) (see Fig. 4). Impacts were mainly due to the plastics used for the window/glass trigger bottle (a.o. polyethylene terephthalate) and the blow moulding process of the plastic bottle.

The impact category strongly affected by ingredients sourcing was natural land transformation (59 %), which was mainly driven by the mixed sourced (palm or coconut and fossil resources) surfactant.

Transport had the largest contribution to photochemical oxidant formation (47 %) and ozone depletion, in particular transport to retailer.

Product manufacture and the end of life phases had the lowest contribution towards the total environmental impacts when compared with the other life cycle stages (see Additional file 1: Table S31).

The cumulative energy demand was 0.24 MJ, of which two-thirds could be attributed to packaging (see Table 2).

#### Identification of relevant impact categories

As shown in Fig. 5, for a window glass trigger spray, the most relevant impact categories relative to the reference were fossil depletion, climate change (the effect on both human health and ecosystem quality), particulate matter formation, natural land transformation and agricultural land occupation. The main factor driving climate change, fossil depletion, and particulate matter formation was packaging, more specifically the resin used for the PET bottle. Agricultural land occupation impacts were also due to packaging, in particular the solid bleached board. Natural land transformation impacts were driven by the oleochemical fraction of the mixed sourced surfactants.

### Bathroom trigger spray

#### Environmental impact of the life cycle stages

Figure 4 shows that for a bathroom trigger spray, the impact categories strongly affected by ingredients sourcing were natural land transformation (93 %), agricultural land occupation (64 %), and water depletion (54 %), mainly driven by the oleochemically (palm or coconut resources) sourced surfactant. Sourcing and production of citric acid had the largest impact on marine eutrophication (73 %), freshwater eutrophication (53 %), and ionising radiation (53 %).

Transport had the largest contribution to terrestrial acidification (56 %), photochemical oxidation formation (47 %), ozone depletion (47 %), particulate matter formation (39 %), and urban land occupation (38 %), in particular transport to retailer.

Packaging had the largest contribution to fossil depletion (51 %), in particular the plastics (e.g. polyethylene) used for the trigger spray bottle. Sources of climate change impacts were evenly spread amongst ingredients sourcing, packaging, and transport.

Product manufacture and the end of life phases had the lowest contribution towards the total environmental impacts when compared with the other life cycle stages (see Additional file 1: Table S32).

The cumulative energy demand was 0.22 MJ, of which 0.10 MJ could be attributed to packaging and 0.08 MJ to the ingredients sourcing (see Table 2).

#### Identification of relevant impact categories

For the bathroom trigger spray investigated, the most relevant impact categories relative to the reference were fossil depletion, natural land transformation, climate change, particulate matter formation, and agricultural land transformation (see Fig. 5). The main factor driving fossil depletion was packaging, more specifically the PE material used in the HDPE bottle. Natural land transformation and agricultural land occupation impacts were driven by the surfactant, whereas impacts on particulate matter formation were mainly due to transport to the retailer. Climate change impacts were a result of the production of the plastics for the bottle (HDPE), the blow moulding process of the plastic bottle, citric acid, and transport to the retailer.

### Acid toilet cleaner

#### Environmental impact of the life cycle stages

Figure 4 shows that for the representative acid toilet cleaner, the impact categories that are strongly affected by the sourcing of ingredients were natural land transformation (i.e. 83 % of all impacts on natural land transformation could be attributed to ingredients sourcing), water depletion (58 %), and ozone depletion (50 %). These impacts were mainly driven by the oleochemical fraction of the mixed sourced surfactants and formic acid production.

Transport had the largest contribution to photochemical oxidation formation (52 %), particulate matter formation (43 %), and urban land occupation (42 %). Particularly, the transport to the retailer was relevant here.

Packaging had the largest contribution on agricultural land occupation (65 %), freshwater eutrophication (51 %), ionising radiation (50 %), and fossil depletion (46 %), in particular the plastics (e.g. polyethylene) used for the toilet cleaner bottle.

Sources of climate change impacts were spread amongst packaging and ingredients sourcing, and to a lesser extent transport. Product manufacture and the end of life phases had the lowest contribution towards the total environmental impacts when compared with the other life cycle stages (see Additional file 1: Table S33).

The cumulative energy demand was 1.09 MJ, of which 0.52 MJ could be attributed to packaging and 0.36 MJ to the sourcing of ingredients (see Table 2).

#### Identification of relevant impact categories

For an acid toilet cleaner, the most relevant impact categories relative to the reference were fossil depletion, climate change (the effect on both human health and ecosystem quality), particulate matter formation, natural land transformation, and agricultural land occupation (see Fig. 5). The main factor driving fossil depletion was packaging, more specifically the PE material used in the HDPE bottle. Natural land transformation impacts were driven by the surfactant. Impacts on agricultural land occupation were mainly due to the solid bleached board. Particulate matter formation impacts were mostly due to transport to the retailer. Climate change impacts were a result of the production of the plastics for the bottle (HDPE), the blow moulding process, transport to retailer, and formic acid production.

### Bleach toilet cleaner

#### Environmental impact of the life cycle stages

Figure 4 shows that for bleach toilet cleaners, impact categories strongly affected by ingredients sourcing were natural land transformation (97 %) and agricultural land occupation (83 %). Both impact categories were mainly driven by the oleochemically (palm or coconut resources) sourced surfactant.

Transport had the largest contribution to ozone depletion (63 %), particulate matter formation (61 %), photochemical oxidation formation (47 %), terrestrial acidification (46 %), and urban land occupation (48 %), in particular transport to retailer.

Packaging had the largest contribution on fossil depletion (51 %), in particular the plastics (e.g. polyethylene) used for the toilet cleaner bottle. Sources of climate change impacts were spread amongst packaging and transport and to a lesser extent ingredients sourcing.

Product manufacture and the end of life phases had the lowest contribution towards the total environmental impacts when compared with the other life cycle stages (see Additional file 1: Table S34).

The cumulative energy demand was 1.28 MJ, and approximately half of that demand could be attributed to packaging (0.63 MJ) and a quarter to the sourcing of ingredients (0.28 MJ) (see Table 2).

#### Identification of relevant impact categories

Figure 5 shows that for a bleach toilet cleaner, the most relevant impact categories relative to the reference were fossil depletion, climate change (the effect on both human health and ecosystem quality), particulate matter formation, natural land transformation, and agricultural land occupation. The main factor driving fossil depletion was packaging, more specifically the PE material used in the HDPE bottle. Natural land transformation and agricultural land occupation impacts were driven by the surfactant, whereas impacts on particulate matter formation were mainly due to transport to the retailer. Climate change impacts were a result of the production of the plastics for the bottle (HDPE), the blow moulding process, transport to retailer, and sodium hypochlorite.

## Discussion

The compilation of life cycle studies in this article shows the environmental impacts of six types of detergents, with exclusion of the human and ecotoxicity impacts (see “System boundaries”). The results are not presented for comparison between products—after all, their intended uses are different in most cases—but to show a number of common observations across the six product categories and to illustrate how the results have helped to inform the choice of Charter ASP criteria. In this section, the sensitivities and limitations of the LCA models used are discussed, the interpretation of the results is addressed, and a description is provided of how the LCA results have been used regarding the development of Charter ASPs.

### Limitations

For the compiled LCAs, environmental and human toxicity impacts were out of the scope. However, to qualify for the Charter Advanced Sustainability Profile logo, member companies must be able to confirm environmental safety of their individual products, in addition to meeting all the other ASP criteria. Hence, environmental and human safety aspects need to be addressed through risk assessment. The approach is described in “System boundaries”.

Water use data associated with many inventories are of poor quality; the water inventory does not distinguish between sources of water or water quality. This should be remembered when interpreting the findings of the study.

Additionally, the life cycle inventories for surfactants, whilst the best available, were over 15 years old and there were no adequate data relating to direct land use change. However, the source of the surfactant (palm kernel, coconut, or fossil resources) directly influences the importance of land use impact categories, especially natural land transformation. Moreover, for compliance with the WRI GHG protocol, ILCD and ISO 14040/44, any direct land use change occurring in the previous 20 years should be considered for above and below ground biomass and for soil organic matter (differentiated for peat and mineral soil). Therefore, we took into account the biogenic fraction of carbon dioxide emissions, both the uptake and release. As the cleaning products are short cyclic, the balance of uptake and release is zero. Due the limited availability of adequate data, the results for impact categories relating to direct land use change and its associated GHG emissions were compromised and must be interpreted with caution.

In all of the underlying LCA studies, choices and assumptions were made that could affect the results. Based on the results of the contribution analysis, we selected important variables per product category to perform a sensitivity analysis. Depending on the product category, these were product dosage, water consumption, wash temperature, energy source for heating the water, surfactant type, packaging material, transport distance, or the method for the end of life recovery (recycled content versus ‘closed loop approximation’, i.e. the benefits and burdens associated with recycling and energy recovery from incineration fall within the scope of the study). The results of the sensitivity analysis did not reveal new insights or changed the hotspots.

### Drivers of the environmental impacts

It can be concluded that in general, the variables that mainly drive the results for manual dishwashing detergents and laundry detergents were the water temperature, the water consumption (for manual dishwashing detergents), the product dosage (for laundry detergents), and the choice and amount of surfactant. By contrast, for bathroom trigger sprays, and acid and bleach toilet cleaners, the driving factors were the plastic packaging, transportation to the retailer, and specific ingredients. Additionally, the type of surfactant was important for bleach toilet cleaners. For window glass trigger sprays, the driving factors were the plastic packaging and the type of surfactant, and the ingredients were of less importance.

Regarding the drivers of the environmental impacts, trade-offs exist between impact categories. Other studies found as well that trade-offs can occur and accordingly that there is no single product environmentally superior on all environmental indicators. Afise, the French detergent association, and P&G performed a comparative LCA to assess the impact of three market-relevant kitchen cleaning products: kitchen cleaning wipes, kitchen cleaning spray, and a liquid household cleaner (LHC) product in a bottle [13]. They found among others that the spray and wipe product consume significantly lower water quantities compared to the LHC product, and that spray or LHC produce less household waste than wipes.

Several studies illustrate that substantial impacts are caused in the consumer-use phase. Koehler and Wildbolz showed for nine home care and personal hygiene products that the impact of these products on the environment would be reduced substantially if consumers could be encouraged to apply only correct product dosages and low water temperatures during product application [14]. Additionally, a study by Stamminger and colleagues showed that the average water consumption for manual dishwashing increases if the load to be cleaned is divided into smaller portions, from on average 103 l for 12 place settings in one go, to more than 121 l six times for 2 place settings [6]. Fuss et al. formulated the best practice tips for manual dishwashing and studied whether they can be used to save resources by affecting behavioural changes [15]. The researchers focussed on common household conditions, such as large amounts of dishes, and observed a reduction in the use of resources when the best practice tips were applied, that is, around 60 % less water, 70 % less energy, and 30 % less detergent. Consumers’ attitude towards best practice tips was generally positive.

Regarding the environmental impacts of other life cycle stages than the consumer-use phase, Koehler and Wildbolz also confirmed that to cut down the energy and materials required for packaging, production and transport, manufacturers should produce detergents in concentrated form [14]. Different waste disposal or recycling options have little effect on environmental impact.

### Practical implications

The LCAs described in the present article were performed as a quantitative basis for the development of Advanced Sustainability Profiles of A.I.S.E.’s Charter for Sustainable Cleaning. The LCAs demonstrated which parameters are the critical ones to be addressed—e.g. in the case of toilet cleaners these were packaging, transport, and optimal use of the product [16]. Using these LCA findings as a starting point, A.I.S.E. determined thresholds, such as a maximum level of packaging materials per job, set a minimum level of recycled/sustainably sourced content in primary and secondary packaging, and provided on-pack guidance towards the most sustainable product use. The criteria are ambitious, but achievable by all market players (see also Fig. 2). Implicit in the ASP criteria is that a product must deliver an acceptable level of performance. Companies must be able to provide evidence to ensure that the product’s performance is acceptable and consistent with the claims made on the product.

Since the Charter was launched in 2005, more than 200 companies have committed to it, representing about 90 % of the total production output for Europe [17]. In the Charter’s first seven full years of operation, verified returns from companies demonstrate how the Charter member’s efforts continue to yield positive results across Europe. The energy consumed per tonne of production was reduced by 18 %, the CO_2_ emitted per tonne of production by 21 %, and the waste per tonne of production by 6 % [1]. In 2013, there were 820 million products with an ASP logo sold [18].

Moreover, the findings from the Charter will also be used by A.I.S.E. as input for the pilot on the Product Environmental Footprint (PEF) [17]. The European Commission and Joint Research Center (JRC) have jointly developed this harmonised methodology for the calculation of the environmental footprint of products. A.I.S.E. is leading the pilot on household liquid laundry detergents over the period 2013–2016 with the objective of developing workable product category rules (PEFCRs) to guide the PEF calculations.

## Additional file

10.1186/s12302-015-0055-4 In the Supplement Material section, the detergent product formulations, inventory data used to model the detergents and the packaging, key assumptions used in the study, the LCIA method structure, and aggregated midpoint results are provided.
