# Supplementary material for: A compilation of life cycle studies for six household detergent product categories in Europe: the basis for product-specific A.I.S.E. Charter Advanced Sustainability Profiles
Source: Environ Sci Eur. 2015 Oct 5;27(1):23. doi: 10.1186/s12302-015-0055-4 (PMC5044932; doi:10.1186/s12302-015-0055-4)
Supplement: Supplementary file 1 — 10.1186/s12302-015-0055-4 In the Supplement Material section, the detergent product formulations, inventory data used to model the detergents and the packaging, key assumptions used in the study, the LCIA method structure, and aggregated midpoint results are provided. [file 12302_2015_55_MOESM1_ESM.docx]

Supporting information

# A compilation of life cycle studies for six detergent categories in Europe: the basis for product specific A.I.S.E. Charter Advanced Sustainability Profiles

Laura Golsteijn, Rimousky Menkveld, Henry King, Christine Schneider, Diederik Schowanek, Sascha Nissen

**Contents**

[1 Life Cycle Inventory 2](#_Toc408489363)

[1.1 Manual dishwashing detergents 2](#_Toc408489364)

[1.2 Powder laundry detergents 4](#_Toc408489365)

[1.3 Window glass trigger spray 6](#_Toc408489366)

[1.4 Bathroom trigger spray 8](#_Toc408489367)

[1.5 Acid toilet cleaners 9](#_Toc408489369)

[1.6 Bleach toilet cleaners 10](#_Toc408489370)

[2 Life Cycle Impact Assessment 13](#_Toc408489372)

[2.1 Method structure 13](#_Toc408489373)

[2.2 Aggregated midpoint results 14](#_Toc408489374)

# Life Cycle Inventory

## Manual dishwashing detergents

The major active component in manual dishwashing detergents is a surfactant mix, which contributes to food residue removal. Other components of detergents may include solubility enhancers, preservatives, perfumes, dyes and opacifiers. Manual dishwashing detergents in the marketplace have a broad range of surfactant level (from 10 - 35%). For this study a rather dilute product with 14% surfactant was selected, as this was currently considered to be most representative in the main markets across Europe. Table S1 shows the product formulation of a manual dishwashing detergent. For more information about the frame formula please see **Table S2**.

**Table S1** Manual dishwashing detergent product formulation.

| **Manual dishwashing product formulation** | **Concentration** |
| --- | --- |
| Softened Water | 83-85 % |
| Ethanol denaturated | < 0.1% |
| Phenoxyethanol | < 1% |
| Propylene Glycol | < 0.1% |
| Surfactant system  (anionic – non-ionic)* | 13.85% |
| NaOH | < 0.2% |
| NaCl | < 2% |
| Perfume | < 0.5% |
| Dye (2 types) | < 0.1% |
| Preservatives | < 0.1% |

* The surfactant system modelled here is AES, with 100% oleochemical origin of the fatty alcohol part (i.e. mix of palm kernel and coconut oil). A petrochemical equivalent exists as well.

**Table S2** shows some of the inventory data used to model the manual dishwashing detergent. The datasets for the packaging materials were taken from the Ecoinvent database.

**Table S2** Ecoinvent data inventory for a manual dishwashing detergents frame formula.

| A manual dishwashing detergent product formulation | Ecoinvent data |
| --- | --- |
| **Water** | RER: water, completely softened, at plant |
| **Ethanol denatured** | RER: ethanol from ethylene, at plant |
| **Phenoxyethanol** | RER: ethylene glycol, at plant |
| **Propylene glycol** | RER: propylene glycol, at plant |
| **Surfactant (anionic-non-ionic)** | RER: ethoxylated alcohols* |
| **NaOH** | RER: sodium hydroxide, 50% in H2O, production mix, at plant |
| **NACI** | RER: sodium chloride, powder, at plant |
| **Perfume** | Empty process |
| **Dye (2 types)** | Empty process |
| **Preservatives** | Empty process |

* Alcohol ethoxylates (AE) with two degrees of ethoxylation AE3 and AE7, 1/6 mix of petrochemical, palm kernel oil, coconut oil

**Table S3** shows the primary and secondary packaging materials used for a one litre bottle of manual dishwashing detergent. The secondary packaging (i.e. cardboard box) consist of 80% recycled material.

**Table S3** Primary & secondary packaging for 1 litre bottle

| **Packaging (Primary& Secondary)** | **Material** | **Weight** |
| --- | --- | --- |
| Bottle | Polyethylene terephthalate (PET) | 43.7g |
| Cap | Polypropylene (PP) | 3.8g |
| Cardboard box | Solid unbleached cardboard | 26g* |

* The weight is allocated per bottle based on the number in a case.

**Table S4** shows the key assumptions used in the study. A sensitivity analysis will be carried out on variables that have a large contribution on the environmental impact.

**Table S4** Key assumptions

|  | **Reference** | **Full sink** | **Direct application** |
| --- | --- | --- | --- |
| **Functional unit** | A.I.S.E. | 4 place settings***** | 4 place settings***** |
| **Reference flow** | Based on Stamminger et al. (2007) | 8 ml | 12 ml |
| **Water consumption** | Stamminger et al. (2007) | 7.5 l | 15 l |
| **Energy for water heating**** | P&G measured data | 0.27 kWh | 0.30 KWh |
| **Energy source for water heating** | Consumer studies | Electricity | Electricity |
| **Transport ingredients** | A.I.S.E. based on P&G data | Renewable part in surfactants 8000 km (boat)  Other ingredients 2000 km (truck) | Renewable part in surfactants 8000 km (boat)  Other ingredients 2000 km (truck) |
| **Transport retail** | A.I.S.E. based on P&G data | 1200 km lorry | 1200 km lorry |
| **Waste emissions from product manufacture** | A.I.S.E | Very low (process efficiency of 99.9%) | Very low (process efficiency of 99.9%) |
| **Waste water treatment************* | Based on EU Statistics | 100% connection to secondary treatment | 100% connection to secondary treatment |
| **Recycling rates solid waste** | Eurostat (2012) | Paper & board 83.2%  Plastic 31.9% | Paper & board 83.2%  Plastic 31.9% |
| **Solid waste treatment** | Eurostat (2012) | Landfill 65.3%  Incineration 34.7% | Landfill 65.3%  Incineration 34.7% |

* Selected to be similar in concept to the Automatic Dishwashing study, but lower amount of items to wash (meal of 1 family).

** The water temperature is based on the maximum temperature people can stand comfortably with bare hands (45 ˚C). This value is somewhat lower for direct application since it is assumed the temperature is a mix between warm cleaning and cold rinsing.

*** Removal rates of substances during secondary wastewater treatment was provided by P&G.

Place setting specifications were taken from Stamminger et al. (2007). One place setting consists of the pieces shown in Table S5.

**Table S5** Place setting specification

| **Item description** | **Diameter/volume/length** | **Shape/Style** |
| --- | --- | --- |
| **Dinner plate** | 260 mm | Arzberg 8500 |
| **Soup plate** | 230 mm | Arzberg 1382 |
| **Dessert dish** | 190 mm | Arzberg 8500 |
| **Cup** | 0,21 L | Arzberg 1382 |
| **Saucer** | 140 mm | Arzberg 1382 |
| **Glass** | 250 ml/ 60 mm | Beaker/ Tall form/ Without drain |
| **Fork** | 184 mm | WMP “Berlin” |
| **Soup spoon** | 195 mm | WMP “Berlin” |
| **Knife** | 203 mm | WMP “Berlin” |
| **Teaspoon** | 126 mm | WMP “Berlin” |
| **Dessert spoon** | 156 mm | WMP “Berlin” |

## Powder laundry detergents

**Table S6** shows the generic product formulation of a tablet and compact powder laundry detergent for the European market (based on information provided by A.I.S.E.).

**Table S6** Tablet powder product formulation.

| **Product formulation** | **Tablet** | **Powder** |
| --- | --- | --- |
| Alkalinity sources | 15-30% | 15-30% |
| Bleach agents | 1-5% | 1-5% |
| Builders | 15-30% | 15-30% |
| Enzymes | 0.2-0.5% | 0.2-0.5% |
| Fragrances | 0.2-0.5% | 0.2-0.5% |
| Optical brighteners | 0.2-0.5% | - |
| Oxidising agents | 5-15% | 5-15% |
| Sequestrants | 1-5% | 1-5% |
| Surfactant system  (anionic – non-ionic)* | 5-15% | 5-15% |
| Water | - | 5-6% |

*The surfactant system modelled here is of a mixed oleochemical (i.e. palm kernel and coconut oil) and petrochemical origin.

**Table S7** and Table S8 show some of the inventory data used to model the solid laundry detergent. The datasets for the packaging materials were taken from the Ecoinvent database.

**Table S7** Ecoinvent data inventory for a tablet powder laundry detergent frame formula.

| A tablet powder laundry detergent product formulation | Ecoinvent data |
| --- | --- |
| **Alkalinity sources** | GLO: sodium carbonate from ammonium chloride production at plant |
| **Bleach percursors** | RER: etylenediamine, at plant  RER: layered sodium silicate, SKS-6, powder at plant |
| **Builders** | RER: polycarboxylates, 40% active substance, at plant  RER: zeolite, powder, at plant  RER: sodium sulphate, powder, at plant |
| **Auxiliaries** | RER: carboxymethyl cellulose, powder at plant  Citric acid*  RER: modified starch, at plant |
| **Enzymes** | Enzymes** |
| **Fragrances** | Empty process |
| **Optical brighteners** | Empty process |
| **Oxidising agents** | Empty process |
| **Water** | RER: water, completely softened, at plant |
| **Surfactant system  (anionic – non-ionic)** | RER: fatty alcohol sulphate mix, at plant***  RER: ethoxylated alcohols, unspecified, at plant**** |

*Citric acid LCI data was provided by Unilever

**Enzymes LCI data was provided by Novozymes

*** Alcohol sulphate (AS) C12-18, 25% mix of petrochemical, palm kernel oil, coconut oil, palm oil

**** Alcohol ethoxylates (AE) with two degrees of ethoxylation AE3 and AE7, 1/6 mix of petrochemical, palm kernel oil, coconut oil

**Table S8** Ecoinvent data inventory for a compact powder laundry detergent frame formula.

| A powder solid laundry detergent product formulation | Ecoinvent data |
| --- | --- |
| **Alkalinity sources** | GLO: sodium carbonate from ammonium chloride production at plant |
| **Bleach percursors** | RER: etylenediamine, at plant  RER: sodium percarbonate, powder at plant  RER: layered sodium silicate, SKS-6, powder at plant |
| **Builders** | RER: polycarboxylates, 40% active substance, at plant  RER: zeolite, powder, at plant  RER: sodium sulphate, powder, at plant |
| **Auxiliaries** | RER: carboxymethyl cellulose, powder at plant  Citric acid*  RER: sodium hydroxide, 50% in H20, production mix  RER: sodium chloride, powder at plant |
| **Enzymes** | Enzymes** |
| **Fragrances** | Empty process |
| **Optical brighteners** | Empty process |
| **Oxidising agents** | RER: sulphuric acid, liquid, at plant |
| **Water** | RER: water, completely softened, at plant |
| **Surfactant system  (anionic – non-ionic)** | RER: fatty alcohol sulphate mix, at plant***  RER: ethoxylated alcohols, unspecified, at plant**** |

*Citric acid LCI data was provided by Unilever

**Enzymes LCI data was provided by Novozymes

*** Alcohol sulphate (AS) C12-18, 25% mix of petrochemical, palm kernel oil, coconut oil, palm oil

**** Alcohol ethoxylates (AE) with two degrees of ethoxylation AE3 and AE7, 1/6 mix of petrochemical, palm kernel oil, coconut oil

**Table S9** shows the primary and secondary packaging materials used for a tablet and compact powder laundry detergent. The secondary packaging (i.e. cardboard box/carton) consists of recycled material (80%).

**Table S9** Primary & secondary packaging for tablet and compact powder laundry detergent at recommended dose

| **Packaging (Primary& Secondary)** | **Material** | **Tablet** | **Powder** |
| --- | --- | --- | --- |
| Flowwrap film | Polypropylene (PP) | 0.35g | - |
| Shrinkwrap | Polyethylene (LDPE) | 0.18g | 0.33g |
| Cardboard box | Corrugated board(80% recycled) | 3*g | 5.9*g |

* The weight is allocated based on the functional unit.

**Table S10** shows the key assumptions used in the study. A sensitivity analysis will be carried out on variables that have a large contribution on the environmental impact.

**Table S10** Key assumptions

|  | **Reference** | **Compact powder** | **Tablet** |
| --- | --- | --- | --- |
| **Functional unit** | A.I.S.E. | 1 wash | 1 wash |
| **Reference flow** | A.I.S.E | 81.5 g | 63.8 g |
| **Use phase** | A.I.S.E | 40°C (0.70 kWh) electricity  60 l water | 40°C (0.70 KWh) electricity  60 l water |
| **Transport ingredients to product manufacturing site** | A.I.S.E. based on P&G data | Renewable part in surfactants 8000 km (boat)  Other ingredients 2000 km (truck) | Renewable part in surfactants 8000 km (boat)  Other ingredients 2000 km (truck) |
| **Waste emissions from product manufacture** | A.I.S.E | Very low (process efficiency of 99.9%) | Very low (process efficiency of 99.9%) |
| **Transport retail** | A.I.S.E. based on P&G data | 1200 km lorry | 1200 km lorry |
| **Waste water treatment** | Based on EU Statistics | 100% connection to secondary treatment | 100% connection to secondary treatment |
| **Recycling rates solid waste** | Eurostat (2012) | Paper & board 83.2%  Plastic 31.9% | Paper & board 83.2%  Plastic 31.9% |
| **Solid waste treatment** | Eurostat (2012) | Landfill 65.3%  Incineration 34.7% | Landfill 65.3%  Incineration 34.7% |

## Window glass trigger spray

**Table S11** shows the product formulation of a window glass trigger spray for the European market (based on information provided by Henkel). For more information about the frame formula please see **Table S12**.

**Table S11** Window glass trigger spray product formulation.

| **Window/Glass trigger spray formulation** | **Concentration** |
| --- | --- |
| Water, demineralised | 93. % |
| Ethanol | <5% |
| Propylene glycol monobutylether | 1% |
| Anionic surfactant system * | <1% |
| Perfume, citral | <1% |

* The anionic surfactant system modelled here is FAS, (65% renewable and 35% inorganic origins) and SAS, (68% petrochemical and 32% inorganic origins).

**Table S12** shows the primary and secondary packaging materials used for a 750 ml window glass trigger spray. The secondary packaging (i.e. cardboard box/carton) consists of recycled material (80%).

**Table S12** Primary & secondary packaging for a 750 ml window glass trigger spray bottle.

| **Packaging (Primary& Secondary)** | **Material** | **Weight (grams)** |
| --- | --- | --- |
| Bottle | PET | 34 |
| Trigger | Polypropylene (PP) & other plastics | 31 |
| Label | paper | 2 |
| Cardboard box | Corrugated board(80% recycled) | 21.5* |

* The weight is allocated per bottle based on the number in a case

**Table S13** shows the inventory data used to model the window glass trigger spray. The datasets for the packaging materials were taken from the Ecoinvent database.

**Table S13** Ecoinvent data inventory for a window glass trigger spray frame formula.

| A window glass trigger spray product formulation | Ecoinvent data |
| --- | --- |
| **Surfactant** | RER: fatty alcohol sulphate, mix, at plant* |
| **Ethanol** | RER: ethanol from ethylene, at plant |
| **Propylene glycol** | RER: propylene glycol, liquid, at plant |
| **Ethylene glycol** | RER: ethylene glycol, at plant |
| **Dye** | Empty process |
| **Fragrance** | Empty process |
| **Demin water** | RER: water, completely softened, at plant |

* Alcohol sulphate (AS) C12-18, 25% mix of petrochemical, palm kernel oil, coconut oil, palm oil

**Table S14** shows the key assumptions used in the study. A sensitivity analysis will be carried out on variables that have a large contribution on the environmental impact.

**Table S14** Key assumptions

|  | **Reference** | **Assumptions** |
| --- | --- | --- |
| **Functional unit** | A.I.S.E. | 1 m^2^ |
| **Reference flow** | A.I.S.E | 10 ml |
| **Transport ingredients to product manufacturing site** | A.I.S.E. based on P&G data | Renewable parts of surfactants 8000 km (boat)  Other ingredients 2000 km (truck) |
| **Waste emissions from product manufacture** | A.I.S.E | Very low (process efficiency of 99.9%) |
| **Transport retail** | A.I.S.E. based on P&G data | 1200 km lorry |
| **Recycling rates solid waste** | Eurostat (2012) | Paper & board 83.2%  Plastic 31.9% |
| **Solid waste treatment** | Eurostat (2012) | Landfill 65.3%  Incineration 34.7% |

## Bathroom trigger spray

**Table S15** shows a representative product formulation of a bathroom trigger spray for the European market (based on information provided by Unilever). For more information about the frame formula please see **Table S16**.

**Table S15** Bathroom trigger spray product formulation

| **Bathroom trigger spray formulation** | **Concentration** |
| --- | --- |
| Surfactant (anionic)* | 1-3% |
| Citric acid/citrate | 2-5% |
| Minors (perfume, colorants, preservatives, etc) | 1-2% |
| Water | ≥ 90% |

*The surfactant system modelled here is Alcohol Ethoxylates (AE) with 100% oleochemical origin of the fatty alcohol part (i.e. mix of palm kernel and coconut oil).

**Table S16** shows the inventory data used to model the bathroom trigger spray. The datasets for the packaging materials were taken from the Ecoinvent database.

**Table S16** Data inventory for a bathroom trigger spray frame formula

| A bathroom trigger spray product formulation | Ecoinvent data |
| --- | --- |
| Surfactant | RER: ethoxylated alcohols, unspecified, at plant* |
| Citric acid | Citric acid** |
| Sodium citrate | Citric acid** |
| Opacifier polymer dispersion | RER: acrylic acid, at plant |
| Linear polyacrylic acid | RER: acrylic acid, at plant |
| Sodium cumene sulphonate | RER: alkylbenzene sulfonate, linear, petrochemical, at plant |
| Fragrance | Empty process |
| 1,2 Benzisothiazolin 3-one | RER: toluene, liquid, at plant |
| Silicone antifoam | RER: silicone product, at plant |
| Demin water | RER: water, completely softened, at plant |

* Alcohol ethoxylates (AE) with two degrees of ethoxylation AE3 and AE7, 1/2 mix of palm kernel oil and coconut oil

** Citric acid LCI data was provided by Unilever

**Table S17** shows the primary and secondary packaging materials used for a 750 ml bathroom trigger spray bottle. The secondary packaging (i.e. cardboard box/carton) consists of recycled material (80%).

**Table S17** Primary & Secondary packaging for a 750 ml trigger spray bottle

| **Packaging (Primary& Secondary)** | **Material** | **Weight (grams)** |
| --- | --- | --- |
| Bottle (750ml) | High-density polyethylene (HDPE) | 48 |
| Trigger | Polypropylene (PP) & other plastics | 21.7 |
| Label | Paper | 1.5 |
| Cardboard box | Solid unbleached cardboard (80% recycled content) | 34.1* |

* The weight is allocated per bottle based on the number in a case.

**Table S18** shows the key assumptions used in the study. A sensitivity analysis will be carried out on variables that have a large contribution on the environmental impact.

**Table S18** Key assumptions

|  | **Reference** | **Assumptions** |
| --- | --- | --- |
| **Functional unit** | A.I.S.E. | 1 m^2^ |
| **Reference flow** | A.I.S.E | 10 ml |
| **Transport ingredients to product manufacturing site** | A.I.S.E. based on P&G data | Renewable parts of surfactants 8000 km (boat)  Other ingredients 2000 km (truck) |
| **Waste emissions from product manufacture** | A.I.S.E | Very low (process efficiency of 99.9%) |
| **Transport retail** | A.I.S.E. based on P&G data | 1200 km lorry |
| **Recycling rates solid waste** | Eurostat (2012) | Paper & board 83.2%  Plastic 31.9% |
| **Solid waste treatment** | Eurostat (2012) | Landfill 65.3%  Incineration 34.7% |

## Acid toilet cleaners

Table S19 shows the generic product formulation of an acid toilet cleaner for the European market (based on information provided by Henkel). For more information about the frame formula please see Table S20.

**Table S19** Acid toilet cleaner product formulation.

| **Acid based toilet cleaner** | **Concentration** |
| --- | --- |
| Formic acid | 5-10% |
| Surfactant (anionic)* | 0-5% |
| Thickener | 0-1% |
| Perfume | 0-1% |
| Dye stuff | 0-1% |
| Water | ≥ 83% |

* The surfactant system modelled here is of a mixed oleochemical and petrochemical origin

**Table S20** shows the inventory data used to model the acid toilet cleaner. The datasets for the packaging materials were taken from the Ecoinvent database.

**Table S20** Ecoinvent data inventory for an acid toilet cleaner frame formula.

| A acid toilet cleaner product formulation | Ecoinvent data |
| --- | --- |
| Formic acid | RER: formic acid, at plant |
| Surfactant | RER: fatty alcohol sulphate, mix, at plant* |
| Sodium hydroxide | RER: sodium hydroxide, 50% in H2O, production mix, at plant |
| Fragrance | Empty process |
| Dye | Empty process |
| Water | RER: water, completely softened, at plant |

*Alcohol sulphate (AS) C12-18, 25% mix of petrochemical, palm kernel oil, coconut oil, palm oil

Table S21 shows the primary and secondary packaging materials used for a 750 ml acid toilet cleaner. The secondary packaging (i.e. cardboard box/carton) consists of recycled material (80%).

**Table S21** Primary & secondary packaging for a 750 ml acid toilet cleaner bottle.

| **Packaging (primary& secondary)** | **Material** | **Weight (grams)** |
| --- | --- | --- |
| Bottle | High-density polyethylene (HDPE) | 46 |
| Spout | Polyethylene (PE) | 0.86 |
| Cap, sealings, nozzles | Polypropylene (PP) | 10.4 |
| Label | Paper | 2 |
| Cardboard box | Corrugated board (80% recycled) | 23.5* |

* The weight is allocated per bottle based on the number in a case.

Table S22 shows the key assumptions used in the study. A sensitivity analysis will be carried out on variables that have a large contribution on the environmental impact.

**Table S22** Key assumptions

|  | **Reference** | **Assumptions** |
| --- | --- | --- |
| **Functional unit** | A.I.S.E. | 1 toilet bowl |
| **Reference flow** | A.I.S.E | 50 ml |
| **Transport ingredients to product manufacturing site** | A.I.S.E. based on P&G data | Renewable parts of surfactants 8000 km (boat)  Other ingredients 2000 km (truck) |
| **Waste emissions from product manufacture** | A.I.S.E | Very low (process efficiency of 99.9%) |
| **Transport retail** | A.I.S.E. based on P&G data | 1200 km lorry |
| **Recycling rates solid waste** | Eurostat (2012) | Paper & board 83.2%  Plastic 31.9% |
| **Solid waste treatment** | Eurostat (2012) | Landfill 65.3%  Incineration 34.7% |

##

## Bleach toilet cleaners

**Table S23** shows the product formulation of a bleach toilet cleaner for the European market (based on information provided by Unilever). More information about the frame formulation is provided in **Table S24**.

**Table S23** Bleach toilet cleaner product formulation.

| **Bleach toilet cleaner** | **Concentration** |
| --- | --- |
| Surfactant (anionic)* | 4-6% |
| Sodium hypochlorite | 3-5% |
| Sodium hydroxide | 0.5-2% |
| Minors (perfume, colorants, preservatives, etc) | 1% |
| Water | ≥ 86% |

* The surfactant system modelled here is Alcohol Ethoxy Sulphate, with 100% oleochemical origin of the fatty alcohol part (i.e. mix of palm kernel and coconut oil).

**Table S24** shows the inventory data used to model the bleach toilet cleaner. The datasets for the packaging materials were taken from the Ecoinvent database.

**Table S24** Ecoinvent data inventory for a bleach toilet cleaner frame formula.

| A bleach toilet cleaner product formulation | Ecoinvent data |
| --- | --- |
| Amine oxide | RER: fatty acids, from coconut oil, at plant |
| Sodium Laurate | RER: fatty acids, from coconut oil, at plant |
| Sodium Hypochlorite | RER: sodium hypochlorite, 15% in H2O, at plant |
| Sodium hydroxide | RER: sodium hydroxide, 50% in H2O, production mix, at plant |
| Sodium silicate | RER: layered sodium silicate, SKS-6, powder, at plant |
| Sodium chloride | RER: sodium chloride, powder, at plant |
| Dye | Empty process |
| Fragrance | Empty process |
| Demin water | RER: water, completely softened, at plant |

**Table S25** shows the primary and secondary packaging materials used for a 750 ml bleach toilet cleaner. The secondary packaging (i.e. cardboard box/carton) consists of recycled material (80%).

**Table S25** Primary & secondary packaging for a 750 ml bleach toilet cleaner bottle.

| **Packaging (primary& secondary)** | **Material** | **Weight (grams)** |
| --- | --- | --- |
| Bottle | High-density polyethylene (HDPE) | 42 |
| Spout | Polypropylene (PP) | 8.2 |
| Cap | Polypropylene (PP) | 4.3 |
| Label | Paper | 2.4 |
| Cardboard box | Corrugated board (80% recycled content) | 23.5 |

* The weight is allocated per bottle based on the number in a case.

**Table S26** shows the key assumptions used in the study. A sensitivity analysis will be carried out on variables that have a large contribution on the environmental impact.

**Table S26** Key assumptions

|  | **Reference** | **Assumptions** |
| --- | --- | --- |
| **Functional unit** | A.I.S.E. | 1 toilet bowl |
| **Reference flow** | A.I.S.E | 80 ml |
| **Transport ingredients to product manufacturing site** | A.I.S.E. based on P&G data | Renewable parts of surfactants 8000 km (boat)  Other ingredients 2000 km (truck) |
| **Waste emissions from product manufacture** | A.I.S.E | Very low (process efficiency of 99.9%) |
| **Transport retail** | A.I.S.E. based on P&G data | 1200 km lorry |
| **Recycling rates solid waste** | Eurostat (2012) | Paper & board 83.2%  Plastic 31.9% |
| **Solid waste treatment** | Eurostat (2012) | Landfill 65.3%  Incineration 34.7% |

# Life Cycle Impact Assessment

## Method structure

**Figure S1** Relationship between LCI parameters (left), midpoint indicator (middle), and endpoint indicator (right) in ReCiPe 2009

For more information and downloads (characterisation and normalisation factors), please see http://www.lcia-recipe.net/file-cabinet

|  | (a) |  | (b) |
| --- | --- | --- | --- |
|  | (c) |  | (d) |
|  | (e) |  | (f) |

| CC | Climate change (kg CO_2_ eq) | IR | Ionising radiation (kg U235 eq) | **■** | Ingredients |
| --- | --- | --- | --- | --- | --- |
| OD | Ozone depletion (kg CFC-11 eq) | ALO | Agricultural land occupation (m^2^·yr) | **■** | Manufacture |
| TA | Terrestrial acidification (kg SO_2_ eq) | ULO | Urban land occupation (m^2^·yr) | **■** | Packaging |
| FE | Freshwater eutrophication (kg P eq) | NLT | Natural land transformation (m^2^·yr) | **■** | Transport |
| ME | Marine eutrophication (kg N eq) | WD | Water depletion (m^3^) | **■** | Use Phase |
| POF | Photochemical oxidant formation (kg NMVOC) | MD | Metal depletion (kg Fe eq) | **■** | End of Life |
| PMF | Particulate matter formation (kg PM10 eq) | FD | Fossil depletion (kg oil eq) |  |  |

**Figure S2** Characterised midpoint results per product category: (a) manual dishwashing detergent (full sink approach), (b) tablet laundry detergent, (c) window glass trigger spray, (d) bathroom trigger spray, (e) acid toilet cleaner, and (f) bleach toilet cleaner.

## Aggregated midpoint results

**Table S27** Aggregate midpoint results for a manual dishwashing detergent (full sink)

| **Impact category** | **Unit** | **Ingredients** | **Manufacture** | **Packaging** | **Transport** | **Use phase** | **End of life** |
| --- | --- | --- | --- | --- | --- | --- | --- |
| **Climate change** | kg CO2 eq | 3.58E-03 | 4.41E-04 | 1.72E-03 | 1.48E-03 | 1.63E-01 | 2.76E-03 |
| **Ozone depletion** | kg CFC-11 eq | 3.20E-10 | 3.32E-11 | 9.51E-11 | 2.35E-10 | 8.00E-09 | 1.02E-10 |
| **Terrestrial acidification** | kg SO2 eq | 1.32E-05 | 1.42E-06 | 5.93E-06 | 9.51E-06 | 6.94E-04 | 6.01E-06 |
| **Freshwater eutrophication** | kg P eq | 1.06E-06 | 3.17E-07 | 7.84E-07 | 1.47E-07 | 1.68E-04 | 4.77E-07 |
| **Marine eutrophication** | kg N eq | 5.37E-06 | 9.29E-08 | 5.00E-07 | 5.24E-07 | 4.62E-05 | 8.22E-07 |
| **Photochemical oxidant formation** | kg NMVOC | 1.54E-05 | 7.85E-07 | 4.84E-06 | 1.48E-05 | 3.47E-04 | 7.37E-06 |
| **Particulate matter formation** | kg PM10 eq | 5.38E-06 | 4.48E-07 | 2.05E-06 | 4.03E-06 | 2.21E-04 | 4.11E-06 |
| **Ionising radiation** | kg U235 eq | 6.62E-04 | 2.52E-04 | 5.25E-04 | 1.37E-04 | 1.27E-01 | 4.39E-04 |
| **Agricultural land occupation** | m2a | 1.99E-03 | 4.16E-06 | 4.17E-04 | 5.97E-06 | 2.47E-03 | 2.34E-05 |
| **Urban land occupation** | m2a | 1.43E-05 | 1.06E-06 | 1.28E-05 | 1.57E-05 | 6.48E-04 | 4.98E-05 |
| **Natural land transformation** | m2 | 2.19E-05 | 6.09E-08 | 2.78E-07 | 5.43E-07 | 1.81E-05 | 8.10E-09 |
| **Water depletion** | m3 | 1.02E-04 | 1.76E-05 | 1.41E-05 | 5.80E-06 | 9.79E-03 | 5.84E-05 |
| **Metal depletion** | kg Fe eq | 2.08E-04 | 4.34E-06 | 9.11E-05 | 7.48E-05 | 7.21E-03 | 7.16E-04 |
| **Fossil depletion** | kg oil eq | 1.59E-03 | 1.32E-04 | 8.12E-04 | 5.26E-04 | 4.42E-02 | 5.20E-04 |

**Table S28** Aggregate midpoint results for a manual dishwashing detergent (direct application)

| **Impact category** | **Unit** | **Ingredients** | **Manufacture** | **Packaging** | **Transport** | **Use phase** | **End of life** |
| --- | --- | --- | --- | --- | --- | --- | --- |
| **Climate change** | kg CO2 eq | 5.37E-03 | 6.49E-04 | 1.61E-03 | 2.21E-03 | 1.84E-01 | 5.37E-03 |
| **Ozone depletion** | kg CFC-11 eq | 4.80E-10 | 4.92E-11 | 7.51E-11 | 3.51E-10 | 9.07E-09 | 2.04E-10 |
| **Terrestrial acidification** | kg SO2 eq | 1.98E-05 | 2.07E-06 | 5.28E-06 | 1.42E-05 | 7.83E-04 | 1.20E-05 |
| **Freshwater eutrophication** | kg P eq | 1.58E-06 | 4.63E-07 | 5.28E-07 | 2.21E-07 | 1.89E-04 | 9.52E-07 |
| **Marine eutrophication** | kg N eq | 8.06E-06 | 1.36E-07 | 2.08E-07 | 7.85E-07 | 5.24E-05 | 1.39E-06 |
| **Photochemical oxidant formation** | kg NMVOC | 2.31E-05 | 1.15E-06 | 4.64E-06 | 2.22E-05 | 3.94E-04 | 1.47E-05 |
| **Particulate matter formation** | kg PM10 eq | 8.07E-06 | 6.55E-07 | 1.84E-06 | 6.03E-06 | 2.51E-04 | 8.21E-06 |
| **Ionising radiation** | kg U235 eq | 9.92E-04 | 3.69E-04 | 3.24E-04 | 2.06E-04 | 1.43E-01 | 8.78E-04 |
| **Agricultural land occupation** | m2a | 2.99E-03 | 6.08E-06 | 2.62E-05 | 8.94E-06 | 3.49E-03 | 4.68E-05 |
| **Urban land occupation** | m2a | 2.15E-05 | 1.54E-06 | 6.16E-06 | 2.35E-05 | 8.33E-04 | 9.93E-05 |
| **Natural land transformation** | m2 | 3.29E-05 | 9.01E-08 | 2.01E-07 | 8.13E-07 | 2.10E-05 | 2.03E-08 |
| **Water depletion** | m3 | 1.75E-04 | 3.82E-06 | 7.72E-06 | 8.69E-06 | 1.84E-02 | 1.17E-04 |
| **Metal depletion** | kg Fe eq | 3.12E-04 | 6.19E-06 | 1.06E-04 | 1.12E-04 | 8.17E-03 | 1.43E-03 |
| **Fossil depletion** | kg oil eq | 2.38E-03 | 1.95E-04 | 9.24E-04 | 7.88E-04 | 4.99E-02 | 1.04E-03 |

**Table S29** Aggregate midpoint results for a tablet laundry detergent

| **Impact category** | **Unit** | **Ingredients** | **Formulation** | **Packaging** | **Transport** | **Use phase** | **End of life** |
| --- | --- | --- | --- | --- | --- | --- | --- |
| **Climate change** | kg CO2 eq | 1.69E-01 | 1.77E-02 | 4.56E-03 | 1.12E-02 | 3.91E-01 | 2.05E-02 |
| **Ozone depletion** | kg CFC-11 eq | 1.86E-08 | 8.70E-10 | 3.60E-10 | 1.81E-09 | 1.92E-08 | 8.12E-10 |
| **Terrestrial acidification** | kg SO2 eq | 7.22E-04 | 7.37E-05 | 1.30E-05 | 6.58E-05 | 1.62E-03 | 4.78E-05 |
| **Freshwater eutrophication** | kg P eq | 8.71E-05 | 1.75E-05 | 1.22E-06 | 1.09E-06 | 3.82E-04 | 3.80E-06 |
| **Marine eutrophication** | kg N eq | 1.79E-04 | 4.97E-06 | 3.48E-06 | 3.89E-06 | 1.09E-04 | 3.49E-06 |
| **Photochemical oxidant formation** | kg NMVOC | 5.16E-04 | 3.70E-05 | 1.44E-05 | 1.10E-04 | 8.27E-04 | 5.83E-05 |
| **Particulate matter formation** | kg PM10 eq | 2.96E-04 | 2.33E-05 | 4.56E-06 | 2.90E-05 | 5.17E-04 | 3.28E-05 |
| **Ionising radiation** | kg U235 eq | 5.31E-02 | 1.39E-02 | 4.45E-04 | 1.03E-03 | 3.05E-01 | 3.51E-03 |
| **Agricultural land occupation** | m2a | 5.27E-02 | 2.29E-04 | 1.78E-03 | 4.61E-05 | 6.12E-03 | 1.87E-04 |
| **Urban land occupation** | m2a | 1.27E-03 | 5.57E-05 | 4.19E-05 | 1.22E-04 | 2.07E-03 | 3.95E-04 |
| **Natural land transformation** | m2 | 3.37E-04 | 1.88E-06 | 9.71E-07 | 4.09E-06 | 4.59E-05 | 1.06E-07 |
| **Water depletion** | m3 | 3.17E-03 | 1.44E-04 | 3.82E-05 | 4.50E-05 | 7.09E-02 | 4.66E-04 |
| **Metal depletion** | kg Fe eq | 1.49E-02 | 2.10E-04 | 1.59E-04 | 5.88E-04 | 5.55E-03 | 5.73E-03 |
| **Fossil depletion** | kg oil eq | 5.54E-02 | 4.81E-03 | 1.89E-03 | 4.03E-03 | 1.06E-01 | 4.15E-03 |

**Table S30** Aggregate midpoint results for a compact powder laundry detergent

| **Impact category** | **Unit** | **Ingredients** | **Formulation** | **Packaging** | **Transport** | **Use phase** | **End of life** |
| --- | --- | --- | --- | --- | --- | --- | --- |
| **Climate change** | kg CO2 eq | 1.27E-01 | 1.77E-02 | 7.58E-03 | 1.61E-02 | 3.91E-01 | 2.08E-02 |
| **Ozone depletion** | kg CFC-11 eq | 1.67E-08 | 8.70E-10 | 7.07E-10 | 2.59E-09 | 1.92E-08 | 8.13E-10 |
| **Terrestrial acidification** | kg SO2 eq | 5.69E-04 | 7.37E-05 | 2.16E-05 | 9.32E-05 | 1.62E-03 | 4.79E-05 |
| **Freshwater eutrophication** | kg P eq | 1.20E-04 | 1.75E-05 | 2.35E-06 | 1.55E-06 | 3.82E-04 | 3.80E-06 |
| **Marine eutrophication** | kg N eq | 1.63E-04 | 4.97E-06 | 6.73E-06 | 5.55E-06 | 1.09E-04 | 4.11E-06 |
| **Photochemical oxidant formation** | kg NMVOC | 3.85E-04 | 3.70E-05 | 2.30E-05 | 1.57E-04 | 8.27E-04 | 5.86E-05 |
| **Particulate matter formation** | kg PM10 eq | 2.09E-04 | 2.33E-05 | 7.63E-06 | 4.12E-05 | 5.17E-04 | 3.28E-05 |
| **Ionising radiation** | kg U235 eq | 4.25E-02 | 1.39E-02 | 8.75E-04 | 1.47E-03 | 3.05E-01 | 3.51E-03 |
| **Agricultural land occupation** | m2a | 3.53E-02 | 2.29E-04 | 3.50E-03 | 6.59E-05 | 6.12E-03 | 1.87E-04 |
| **Urban land occupation** | m2a | 1.05E-03 | 5.57E-05 | 8.22E-05 | 1.75E-04 | 2.07E-03 | 3.96E-04 |
| **Natural land transformation** | m2 | 2.91E-04 | 1.88E-06 | 1.91E-06 | 5.84E-06 | 4.59E-05 | 9.64E-08 |
| **Water depletion** | m3 | 2.76E-03 | 1.44E-04 | 7.19E-05 | 6.43E-05 | 7.09E-02 | 4.66E-04 |
| **Metal depletion** | kg Fe eq | 1.02E-02 | 2.10E-04 | 3.12E-04 | 8.41E-04 | 5.55E-03 | 5.73E-03 |
| **Fossil depletion** | kg oil eq | 4.27E-02 | 4.81E-03 | 2.62E-03 | 5.75E-03 | 1.06E-01 | 4.15E-03 |

**Table S31** Aggregate midpoint results for a window glass trigger spray

| **Impact category** | **Unit** | **Ingredients** | **Manufacture** | **Packaging** | **Transport** | **Use phase** | **End of life** |
| --- | --- | --- | --- | --- | --- | --- | --- |
| **Climate change** | kg CO2 eq | 1.15E-03 | 1.28E-04 | 5.61E-03 | 2.03E-03 | - | 9.61E-04 |
| **Ozone depletion** | kg CFC-11 eq | 2.16E-10 | 6.29E-12 | 2.43E-10 | 3.27E-10 | - | 2.71E-12 |
| **Terrestrial acidification** | kg SO2 eq | 3.98E-06 | 5.33E-07 | 1.96E-05 | 1.17E-05 | - | 1.75E-07 |
| **Freshwater eutrophication** | kg P eq | 5.38E-07 | 1.26E-07 | 2.59E-06 | 1.95E-07 | - | 6.00E-09 |
| **Marine eutrophication** | kg N eq | 3.60E-07 | 3.59E-08 | 1.41E-06 | 7.00E-07 | - | 1.30E-06 |
| **Photochemical oxidant formation** | kg NMVOC | 4.82E-06 | 2.67E-07 | 1.66E-05 | 1.98E-05 | - | 3.88E-07 |
| **Particulate matter formation** | kg PM10 eq | 1.42E-06 | 1.68E-07 | 6.67E-06 | 5.18E-06 | - | 7.21E-08 |
| **Ionising radiation** | kg U235 eq | 2.49E-04 | 1.01E-04 | 1.83E-03 | 1.85E-04 | - | 4.75E-06 |
| **Agricultural land occupation** | m2a | 1.74E-04 | 1.65E-06 | 1.32E-03 | 8.34E-06 | - | 2.66E-07 |
| **Urban land occupation** | m2a | 3.87E-06 | 4.03E-07 | 3.69E-05 | 2.22E-05 | - | 2.53E-06 |
| **Natural land transformation** | m2 | 1.68E-06 | 1.36E-08 | 7.31E-07 | 7.37E-07 | - | -2.80E-08 |
| **Water depletion** | m3 | 2.78E-05 | 1.04E-06 | 4.44E-05 | 8.14E-06 | - | 5.97E-07 |
| **Metal depletion** | kg Fe eq | 5.79E-05 | 1.52E-06 | 2.15E-04 | 1.06E-04 | - | 1.11E-06 |
| **Fossil depletion** | kg oil eq | 7.15E-04 | 3.48E-05 | 2.81E-03 | 7.27E-04 | - | 6.14E-06 |

**Table S32** Aggregate midpoint results for a bathroom trigger spray

| **Impact category** | **Unit** | **Ingredients** | **Manufacture** | **Packaging** | **Transport** | **Use phase** | **End of life** |
| --- | --- | --- | --- | --- | --- | --- | --- |
| **Climate change** | kg CO2 eq | 3.12E-03 | 2.11E-04 | 3.29E-03 | 1.97E-03 | - | 7.22E-04 |
| **Ozone depletion** | kg CFC-11 eq | 2.18E-10 | 3.02E-11 | 1.05E-10 | 3.17E-10 | - | 1.85E-12 |
| **Terrestrial acidification** | kg SO2 eq | 1.28E-05 | 1.52E-07 | 1.10E-05 | 1.17E-05 | - | 1.05E-07 |
| **Freshwater eutrophication** | kg P eq | 1.46E-06 | 3.27E-09 | 1.08E-06 | 1.92E-07 | - | 1.76E-09 |
| **Marine eutrophication** | kg N eq | 6.16E-06 | 5.81E-09 | 9.44E-07 | 6.85E-07 | - | 6.19E-07 |
| **Photochemical oxidant formation** | kg NMVOC | 9.43E-06 | 2.05E-07 | 1.16E-05 | 1.94E-05 | - | 2.21E-07 |
| **Particulate matter formation** | kg PM10 eq | 4.35E-06 | 5.00E-08 | 3.72E-06 | 5.12E-06 | - | 4.47E-08 |
| **Ionising radiation** | kg U235 eq | 1.07E-03 | 2.22E-06 | 7.61E-04 | 1.81E-04 | - | 2.24E-06 |
| **Agricultural land occupation** | m2a | 1.74E-03 | 7.95E-08 | 9.62E-04 | 8.07E-06 | - | 1.87E-07 |
| **Urban land occupation** | m2a | 1.16E-05 | 9.26E-08 | 2.09E-05 | 2.14E-05 | - | 1.79E-06 |
| **Natural land transformation** | m2 | 1.34E-05 | 4.65E-08 | 3.40E-07 | 7.18E-07 | - | -1.99E-08 |
| **Water depletion** | m3 | 6.37E-05 | 2.16E-05 | 2.46E-05 | 7.86E-06 | - | 4.15E-07 |
| **Metal depletion** | kg Fe eq | 1.23E-04 | 9.30E-07 | 4.87E-05 | 1.02E-04 | - | 7.54E-07 |
| **Fossil depletion** | kg oil eq | 1.06E-03 | 7.87E-05 | 1.90E-03 | 7.05E-04 | - | 4.24E-06 |

**Table S33** Aggregate midpoint results for an acid toilet cleaner

| **Impact category** | **Unit** | **Ingredients** | **Manufacture** | **Packaging** | **Transport** | **Use phase** | **End of life** |
| --- | --- | --- | --- | --- | --- | --- | --- |
| **Climate change** | kg CO2 eq | 1.35E-02 | 6.14E-04 | 1.66E-02 | 1.10E-02 | - | 3.32E-03 |
| **Ozone depletion** | kg CFC-11 eq | 2.31E-09 | 3.02E-11 | 5.21E-10 | 1.77E-09 | - | 8.35E-12 |
| **Terrestrial acidification** | kg SO2 eq | 5.22E-05 | 2.56E-06 | 5.80E-05 | 6.41E-05 | - | 4.72E-07 |
| **Freshwater eutrophication** | kg P eq | 4.85E-06 | 6.07E-07 | 6.71E-06 | 1.06E-06 | - | 7.73E-09 |
| **Marine eutrophication** | kg N eq | 4.69E-06 | 1.72E-07 | 4.31E-06 | 3.81E-06 | - | 2.77E-06 |
| **Photochemical oxidant formation** | kg NMVOC | 4.15E-05 | 1.28E-06 | 5.70E-05 | 1.08E-04 | - | 9.78E-07 |
| **Particulate matter formation** | kg PM10 eq | 1.77E-05 | 8.07E-07 | 1.94E-05 | 2.83E-05 | - | 2.01E-07 |
| **Ionising radiation** | kg U235 eq | 3.46E-03 | 4.84E-04 | 5.03E-03 | 1.01E-03 | - | 9.58E-06 |
| **Agricultural land occupation** | m2a | 2.47E-03 | 7.93E-06 | 4.62E-03 | 4.52E-05 | - | 8.42E-07 |
| **Urban land occupation** | m2a | 5.47E-05 | 1.93E-06 | 1.04E-04 | 1.20E-04 | - | 8.13E-06 |
| **Natural land transformation** | m2 | 2.79E-05 | 6.54E-08 | 1.68E-06 | 4.00E-06 | - | -9.05E-08 |
| **Water depletion** | m3 | 2.46E-04 | 4.98E-06 | 1.28E-04 | 4.41E-05 | - | 1.88E-06 |
| **Metal depletion** | kg Fe eq | 6.04E-04 | 7.29E-06 | 2.28E-04 | 5.76E-04 | - | 3.39E-06 |
| **Fossil depletion** | kg oil eq | 6.46E-03 | 1.67E-04 | 9.11E-03 | 3.94E-03 | - | 1.92E-05 |

**Table 34** Aggregate midpoint results for a bleach toilet cleaner

| **Impact category** | **Unit** | **Ingredients** | **Manufacture** | **Packaging** | **Transport** | **Use phase** | **End of life** |
| --- | --- | --- | --- | --- | --- | --- | --- |
| **Climate change** | kg CO2 eq | 1.09E-02 | 4.03E-03 | 1.80E-02 | 1.77E-02 | - | 4.70E-03 |
| **Ozone depletion** | kg CFC-11 eq | 9.55E-10 | 3.76E-10 | 3.45E-10 | 2.85E-09 | - | 1.21E-11 |
| **Terrestrial acidification** | kg SO2 eq | 4.64E-05 | 1.03E-05 | 6.27E-05 | 1.04E-04 | - | 6.87E-07 |
| **Freshwater eutrophication** | kg P eq | 6.32E-06 | 2.16E-06 | 5.87E-06 | 1.72E-06 | - | 1.14E-08 |
| **Marine eutrophication** | kg N eq | 1.40E-05 | 6.56E-07 | 2.89E-06 | 6.14E-06 | - | 4.00E-06 |
| **Photochemical oxidant formation** | kg NMVOC | 3.86E-05 | 6.33E-06 | 6.65E-05 | 1.74E-04 | - | 1.44E-06 |
| **Particulate matter formation** | kg PM10 eq | 1.73E-05 | 3.27E-06 | 2.10E-05 | 4.58E-05 | - | 2.92E-07 |
| **Ionising radiation** | kg U235 eq | 3.80E-03 | 1.72E-03 | 4.58E-03 | 1.62E-03 | - | 1.44E-05 |
| **Agricultural land occupation** | m2a | 2.32E-02 | 2.85E-05 | 4.77E-03 | 7.27E-05 | - | 1.22E-06 |
| **Urban land occupation** | m2a | 9.18E-05 | 7.59E-06 | 1.00E-04 | 1.93E-04 | - | 1.17E-05 |
| **Natural land transformation** | m2 | 3.04E-04 | 6.44E-07 | 1.34E-06 | 6.45E-06 | - | -1.31E-07 |
| **Water depletion** | m3 | 1.31E-04 | 1.69E-04 | 1.27E-04 | 7.09E-05 | - | 2.71E-06 |
| **Metal depletion** | kg Fe eq | 1.09E-03 | 3.35E-05 | 1.70E-04 | 9.26E-04 | - | 4.92E-06 |
| **Fossil depletion** | kg oil eq | 3.27E-03 | 1.29E-03 | 1.12E-02 | 6.35E-03 | - | 2.77E-05 |
